# The first 100 patients treated with a new anatomical pre-contoured locking plate for clavicular midshaft fractures

**DOI:** 10.1186/s12891-018-2396-9

**Published:** 2019-01-05

**Authors:** Tania Reisch, Roland S. Camenzind, Reto Fuhrer, Ulf Riede, Naeder Helmy

**Affiliations:** Department of Orthopedic Surgery, Bürgerspital Solothurn, Schöngrünstrasse 38, 4500 Solothurn, Switzerland

**Keywords:** Clavicular fracture, Midshaft, Precontoured plate

## Abstract

**Background:**

Pre-contoured locking plates were recently introduced in the management of clavicular midshaft fractures. These plates may offer advantages such as no necessity for intraoperative bending and reduced plate irritation. The purpose of this study was to review the clinical and radiographical outcome of the first 100 patients treated with a new anatomical pre-contoured locking plate.

**Methods:**

In a retrospective single-center study, 100 consecutive patients (16 female, 84 male) with a median age of 40 years (range 15–82) who underwent surgery for clavicular midshaft fractures with a VariAx locking plate (Stryker Corporation Kalmazoo, MI, USA) between March 2012 and January 2016 were included. Postoperative follow-up was performed until union was clinically and radiographically achieved. Fracture type, surgical time, intraoperative need for contouring the plate, further surgery such as revision or hardware removal and complications were recorded.

**Results:**

One-hundred patients with a dislocated midshaft clavicular fracture with a mean follow-up of 21.9 months (standard deviation 13.2) were included. Ninety-three patients reported normal shoulder function at latest follow-up. Median surgical time was 75.5 min (range, 35–179). In three patients, intraoperative bending of the plate was necessary. In two patients, plates designed for the other side were implanted. Five patients needed revision surgery: One patient with wound healing problems, one patient with a re-fracture after early (13 months) hardware removal and minor trauma, one patient with postoperative shoulder stiffness and two patients with failed osteosynthesis because of surgical implantation fault. One asymptomatic nonunion without further treatment was observed. In 30 patients, the plate was removed after a mean of 17.5 months (SD 4.2) because of subjective plate discomfort.

**Conclusions:**

With this new pre-contoured locking plate, good to excellent intraoperative fit to the anatomical shape of the clavicle can be achieved. The implant seems to be reliable regarding handling and complications. Clinical and radiological results are comparable to results reported in the literature. Hardware removal rate is comparable to other studies with a pre-contoured plate and lower compared to non-pre-contoured.

## Background

Clavicle fractures are common with an average of 2.6% of all fractures and most often occur in the middle third of the clavicle [1]. Even though many of these fractures can be treated conservatively, recent evidence suggests that dislocated fracture types show an increased complication rate including a higher non- or malunion rate and therefore operative treatment of displaced and/or comminuted clavicle midshaft fractures has become more popular in the last decades [2–6]. Due to the distinct anatomy of the clavicle, plate fixation in displaced multifragmentary midshaft fractures can be technically challenging. In the past, reconstruction plates were often used due to their contourability and their lower profile. From a biomechanical point of view, the drawback was a diminished implant rigidity, which could lead to hardware failures such as breaking or bending [3, 7]. Furthermore, implant removal rate because of irritating plates is still high up to 38% [8]. Recently, the anatomic pre-shaped clavicle plates were introduced, to address the above-mentioned issues. They offer a higher biomechanical stability [9] without the need for intraoperative bending [10] and thus facilitate fixation. Moreover, these plates may reduce plate irritation due to the low profile and beveled edges and thus have shown to lead to a decreased hardware removal rate [11, 12]. Postoperative range of motion (ROM) and subjective scores does not seem to be affected by the choice of a contoured or non-contoured plate [12].

In this study, the VariAx Clavicle Locking Plate system (Stryker Corporation Kalmazoo, MI, USA), an anatomically pre-shaped, variable angled locking plate system with various plates designed for the most common clavicle fractures was used. It was designed using a CT Scan Database of 160 clavicles of different size, gender, and ethnicity. Made of titanium alloy and treated with Type II anodization, these plates are designed to carry the loads that are required them while remaining low profile. Kienast et al. [13] compared the plate-to-bone fitting of manually bent reconstruction plates with the precontoured VariAx Clavicle locking plate developed using by an anatomical 3D computed tomography-bone database. These data showed a higher degree of plate fit for the osteosynthesis plate developed by using the anatomic 3D database.

However, it remains unclear if the improved plate-to-bone fit has an impact on the clinical and radiographical outcome. The purpose of this study was to evaluate the clinical and radiological outcomes including the rate of plate prominence, rate of hardware removal and rate of complications as well as intraoperative plate-to-bone fit, postoperative ROM and subjective scores in the first 100 patients that have received an open reduction and internal fixation using a pre-shaped locking plate.

## Methods

This study was approved by the local ethical committee (EKNZ BASEC 2016–00454). Informed consent was obtained from all the patients before surgery. In a retrospective single-center study, 100 patients (16 female, 84 male) with a median age of 40 years (range 15–82) who underwent surgery for clavicular midshaft fractures with a VariAx locking plate between March 2012 and January 2016 were included. The indications for surgery included (1) fractures with greater than 20 mm of clavicle shortening, (2) substantially displaced fracture fragments lacking cortical apposition (3) impending skin compromise and (4) multifragmentary fractures. Exclusion criteria were pathologic fractures. Surgery was performed in most patients (74%) within 3 days after initial trauma depending on operating room capacity. In 25 patients, surgery was performed after 1 or 2 weeks in case of secondary dislocation in the radiological control with initial try of a conservative management. In one case, time to surgery was about 16 months. In this case, a conservative treatment of a clavicle fracture ended up in a symptomatic non- and malunion, so that a surgery with cancellous bone grafting from iliac crest and stabilization trough VariAx plate was indicated.

### Surgical technique

All patients obtained a single-shot antibiotic therapy with a cephalosporin 30 min before surgery. After general anesthesia, patients were positioned in a beach-chair position. The skin incision was either centered or vertical over the fracture with dissection of the platysma. The branches of supraclavicular nerve were identified and protected when possible. The soft tissue was stripped of the subcutaneous surface of the cranial clavicle surface in an epiperiosteal plane. The fracture ends were exposed and debrided of hematoma and interposed soft tissue. Simple or, where possible also multifragmentary fractures were reduced by using clamps and fixed with one or two 2.7 mm lag screws before fixing the fracture with a plate. Where reduction was not possible, fractures were reduced indirectly by bridging the fracture with a plate and then reducing the fragments between the most lateral and most medial fragment and fixing them using an osteosuture (Fiberwire 2.0, Arthrex, Naples, FL, USA). The surgeon chose the plate type intraoperatively depending on subjective best fit. Every surgeon had to describe the anatomical fit of the plate in the surgical report. Good anatomical fit was defined as easy positioning of the plate to the bone, small plate-to-bone distance and no need for bending to achieve this. In all patients, a superior plate was implanted - either standard midshaft (*n* = 54), bridging midshaft (*n* = 38) or plates with lateral extension in fractures with large comminution zone (*n* = 8). The plate was fixed preliminary with clamps. Depending on bone quality, 3.5 mm polyaxial locking screws or non-locking screws were used with at least three bi-cortically placed screws on either side of the fracture. After wound irrigation, the wound was closed in layers using an intraarticular suture with an absorbable suture for skin closure.

### Postoperative care and clinical follow-up

All patients were immobilized with a sling for comfort. Active and passive movement of the affected shoulder with a range of motion (ROM) of maximum 90 degrees in flexion and abduction without weight bearing for 6 weeks was recommended. After 6 weeks, free ROM and free weight bearing as tolerated was allowed. Standard thromboembolic prophylaxis was administered only during hospital stay with low molecular weight heparin. Antalgic therapy was implemented by NSAID and paracetamol.

The first postoperative clinical and radiographical follow-up was performed after 6 weeks. Further follow-up has been performed in symptomatic patients 3 or 6 months postoperatively.

In patients complaining of discomfort by hardware, plate removal was performed at the earliest 18 months postoperative except in one case. Here, the plate was removed after 13 months because of patients urge despite our dissuasion. In all cases, where clinical follow-up ended up after 3 months, the patient was called at a minimum of 12 months postoperative and a standardized questionnaire was filled. In this questionnaire, we asked patients about their subjective shoulder function, the ROM compared to the non-affected shoulder, plate discomfort and ability for work. In these cases, the date of questionnaire completion was recorded as the date of the last follow-up.

### Outcome measures

The following data were collected for all the patients: Demographic information (age, sex, time to last follow-up, age at surgery, affected side, fracture location, the median American Society of Anesthesiologists (ASA) classification, fracture type and classification according to Robinson [14], surgical time, intraoperative need for contouring the plate, further surgery such as revision or hardware removal, complications such as plate discomfort, and subjective shoulder function (Table 1).

**Table 1:** Details of the patients: Demographics

Numbers of patients	100
Sex (male/female)	84/16
Follow-up [m]	22 ± 13.2
Age at surgery [y]	39.5 ± 16.9
Operation on right side	63/100 (63%)
Open fractures	2/100 (2%)
Fracture region	
Median 3/5th	100/100 (100%)
Medial 1/5th	0
Lateral 1/5th	0
Fracture classification (Robinson)	
Type 2 a1.	0
Type 2 a2.	1/100 (1%)
Type 2 b1.	34/100 (34%)
Type 2 b2.	65/100 (65%)
ASA	1.4 ± 0.6

Data presented as mean (standard deviation) or n (%)

*ASA* American Society of Anaesthesiologists

### Statistics

All statistics were analyzed using GraphPad Prism 7.02 (GraphPad, La Jolla, CA, USA). Fisher’s exact test was performed to calculate Odds ratio (OR). A *p*-value < 0.05 was considered to be statistically significant. All results are presented in medians (range) or mean (standard deviation).

## Results

One-hundred patients with dislocated midshaft clavicular fractures with a mean follow-up of 21.9 months (SD 13.2) were included. Eighty-five patients completed at least a 1-year follow-up. Ten patients had a minimal follow-up of 3 or 6 months. Five patients were lost to follow-up: Two patients with multiple trauma died because of complications unrelated to the clavicle fracture within 8 weeks postoperative, one patient returned to prison after surgery, one patient returned to his native country, and in one case, the patient could not be reached.

### Clinical results

From 95 reviewed patients, 93 patients (98%) reported a normal subjective shoulder function at last follow-up. Two patients (2%) complained about slight residual pain during activity.

Ninety-two of these 95 patients showed a symmetrical ROM while 3 patients had a reduced ROM in the affected shoulder compared to the non-affected shoulder: one patient with a shoulder stiffness which was treated with arthroscopic capsulotomy after 5 and 9 months. In the second arthroscopy, Propionibacterium acnes was found and treated with antibiotics and a hardware removal after 1 year showed an implant-associated infection as well. At last follow-up (23 months after clavicle fracture), the patient showed a symmetrical ROM. The other patient with reduced ROM had a non-revised plate dislocation, and the last one had primary osteoarthritis of the shoulder.

Thirty-six patients (38%) complained of plate discomfort. In 5 patients this information was missing because there was no follow-up. In 30 patients (32%), the plate was removed after a median of 17.5 months (range, 12–31). The main reason was subjective plate discomfort.

With the numbers available, female patients had a non-significant 2.063-fold higher risk for plate removal than male (OR 2.063 (CI 95%: 0.740–6.261, *p* = 0.236).

### Radiological results

**Fig. 1:**
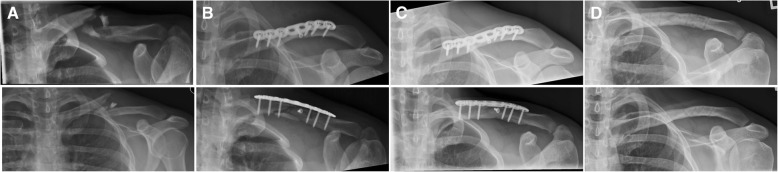
**a** X-ray in two planes of a 25 years old male patient with multifragmentary midshaft clavicular fracture, **b** postoperatively, **c** 5 months after surgery with consolidation of the fracture and **d** 6 weeks follow-up after hardware removal done 12 months after initial surgery shows solid bone union

Standardized radiographs of the clavicle (a.-p. - and tangential view) were performed on the day of the injury, 1 or 2 days postoperatively and in line with the first follow-up after 6 weeks. In the case of no consolidation after 6 weeks and persisting pain, another radiograph has been done at 3 months and/or 1 year postoperatively. A total of 77 patients showed consolidation after a median of 3.5 months (Fig. 1), in one patient an asymptomatic nonunion could be found and in the other 22 patients the information about radiographic consolidation was missing because of no further radiological follow-up due to clinical pain-freedom at 6 weeks or 3 months follow-up (17 patients) or loss of follow-up (the above described 5 patients).

### Intraoperative findings/results

The median surgical time was 75.5 min (range, 35–179). The anatomical fit of the VariAx plate was reviewed in the surgical rapport and was subjectively described as very good with easy positioning, low plate-to-bone distances and no need of intraoperative bending in most rapports. In only three patients (3%) intraoperative bending of the plate was necessary; in two cases (2%) because of anatomical reasons only and in one case (1%) the plate designed for the other side showed better anatomical fit.

### Complications

**Fig. 2:**
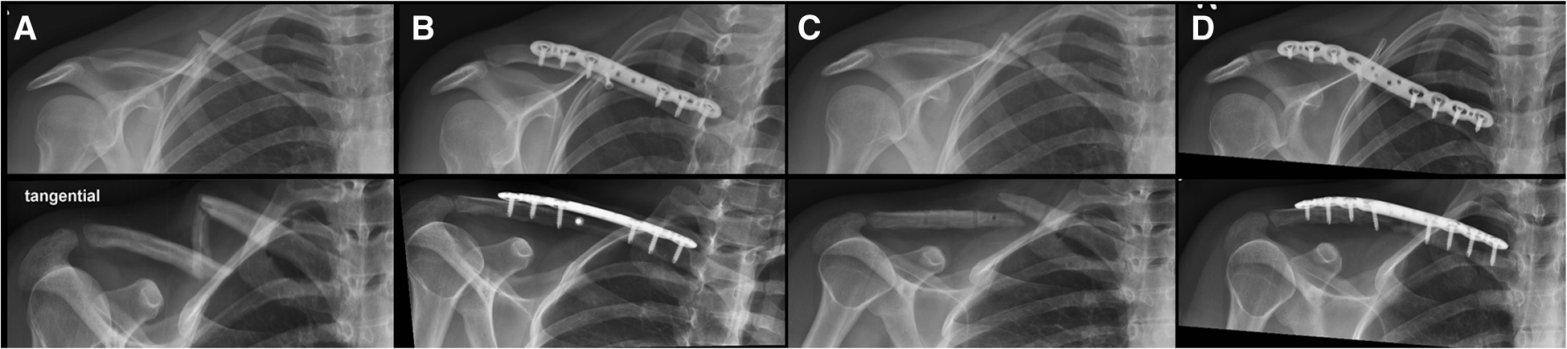
**a** X-ray in two planes of a 32 years old female patient with multifragmentary midshaft clavicular fracture, **b** 5 months after surgery with consolidation of the fracture and **c** 5 days after hardware removal done 14 months after initial surgery showing refracture after minor trauma and **d** 6 weeks after revision surgery with a VariAx locking plate

Five patients (5%) needed revision surgery (Table 2). One patient needed soft tissue revision because of wound healing problem and a non-infected hematoma. One patient needed re-osteosynthesis because of a re-fracture after hardware removal (13 months postoperative) and a minor trauma (Fig. 2). In three patients, failures occurred due to surgical errors where the medial screws were not placed bi-cortically, with subsequent medial plate loosening. One error occurred in experienced and two in less experienced surgeons. Two of these patients needed revision surgery with no further problems in follow-up after revision surgery. One patient was treated for postoperative shoulder stiffness with arthroscopic capsulotomy after 5 and 9 months. One patient developed an asymptomatic nonunion.

**Table 2:** Results

Plate discomfort	36/95 (38%)
Plate removal	30/98 (31%)
Complication systemic	0
Complication local	7/97 (7%)
Revision rate	5/97 (5%)
Nonunion	1/78 (1%)
Normal shoulder function	93/96 (97%)
No pain	94/96 (98%)

## Discussion

Using plates seems to be favorable in the treatment of displaced clavicular midshaft fractures regarding the decreased risk of nonunion by providing higher stability. Regarding implant choice and plate positioning, every system seems to have its own advantages and disadvantages. Superior plate positioning, i.e., provides higher stability regarding the bending forces. Non-pre-contoured plates, i.e. pelvic reconstruction plates, are designed to bend easily at the cost of stability with reported implant failures due to plate weakness. Plate bending of non-contoured plates to achieve an appropriate intraoperative fit of the plate to the variety of human clavicle anatomy is challenging. For experienced surgeons fit accuracy after bending a low profile 3.5 mm pelvic reconstruction plate seems to be better compared to a decent pre-contoured plate (Arthrex Inc., Naples FL, USA) [15]. But the rate of implant related complications like skin irritation or implant prominence still remains high. Pre-contoured plates, therefore, provide biomechanically more stability [9] and may reduce plate irritation and with that lead to less secondary surgery for hardware removal [12, 16, 17]. The VariAx locking plate has been designed 3-dimensionally by using CT-Scan-Database of 160 clavicles, what may improve the anatomical fit to most of the clavicles and so avoid the need for intraoperative bending and reduce the plate irritation. In a comparison study of 5 pre-contoured clavicle plates in 20 embalmed human clavicles, the VariAx plate showed the best anatomical fit of all investigated plates [18]. However, clinical results for this pre-shaped plate are missing.

In our study, we reviewed the first 100 patients treated with pre-shaped VariAx locking plate at our institution and analyzed the clinical and radiological outcomes including the rate of plate prominence, rate of hardware removal and rate of complications as well as intraoperative plate-to-bone fit, postoperative ROM and subjective scores.

Regarding the plate irritation, we still had a high incidence of plate discomfort (36 patients) with a removal rate of 31%. The mean reason was subjective plate discomfort. Our cohort is young and accident mostly happened during sport activity. So, we assume that most patients calculate the risk for complications in a re-operation e.g. plate-removal seems low regarding the benefit of having no more plate irritation especially while sport activity. Furthermore, we suppose, that in some cases the cosmetic aspect assumes a role. This could explain the higher removal rate in female patients which is according to a recent study where female patients are 4 times more likely to undergo hardware removal [16, 19]. Compared to other studies with pre-contoured superior clavicle plates we have a comparable rate of plate discomfort, while the removal rate is still higher (Table 3). So, we obviously complied more often to patients request for plate removal. I.e. VanBeek et al. [12] compared in a retrospective study non-contoured to pre-contoured superior clavicle plate fixation in 52 patients. Patients complained of prominent hardware in 64% in non-contoured plates and 32% in pre-contoured plates with hardware removal in 21 and 10% of the cases respectively. Additionally, the superior positioning of the plate is another risk factor for symptomatic hardware [20].

**Table 3:** Comparison of reported hardware removal rates for midshaft clavicular fractures treated with noncontoured or precontoured plates

Study	Year	Type of plate	Removal rate (%)
Kabak [22]	2004	Noncontoured	39
Chen [7]	2008	Noncontoured	74
Chandrasenan [20]	2008	Precontoured	0
		Noncontoured	60
VanBeek [12]	2011	Precontoured	11
		Noncontoured	21
Lai [23]	2012	Noncontoured	37
Campochiaro [24]	2012	Precontoured	15
Gilde [25]	2014	Noncontoured	9
Ranalletta [11]	2015	Precontoured	13
Rongguang [18]	2016	Noncontoured	66
		Precontoured	45
Our study	2018	Precontoured	31

Regarding intraoperative handling of the VariAx plate most surgeons reported good anatomical fit and even less experienced surgeons performed surgery in secure way. Bending of the plate was necessary in only 3 cases to achieve perfect fit to the specific patient’s anatomy. Although bending of the plate is usually not necessary, it can be performed in a secure way when the surgeon avoids sharp bends, reverse bends or bending the device at a screw hole. We had no plate breakage, but a failure of osteosynthesis with loosening of the medial screws and further plate dislocation due to surgeon’s technical fault. Our complication rate is low; only five patients needed revision surgery of which only one patient had a deep, implant-associated infection. No neurovascular complication was observed in any of the 100 patients. In a comparison study of conventional to pre-contoured plates, 2 of 15 patients treated with a conventional plate had to be revised because of plate breakage [17]. Reconstruction plate furthermore showed a failure rate of 12.6% and revision rate of 6.3% in 111 patients [8]. Böstman et al. [21] evaluated the pitfalls of plate fixation of midclavicular fractures in 1997. They showed a total complication rate of 23% in 103 patients with major complication like deep infection, plate breakage, nonunion and refracture after plate removal. The reoperation rate was about 14% including hardware removal.

In conclusion, we can say that the VariAx locking plate is a secure surgery tool for clavicle midshaft fractures with good anatomical fit and a low complication rate. Regarding the costs, in our hospital setting the VariAx plate is one third more expensive than a regular LCP reconstruction plate with 4 locking and 2 non locking screws in each plate.

The clinical outcome was very good with normal subjective shoulder function, symmetrical ROM and return to previous level of acivity in 93 of 95 patients at last follow-up. These results are comparable to other scores in the literature where Constant scores between 86.5 and 96.1 and DASH scores between 4.3 and 5.2 are reported [3, 6].

Value of radiological outcome using the VariAx plate in our first 100 patients is diminished because of lower follow-up rate. From 78 radiographs at last follow-up, 77 showed consolidations and only one patient showed an asymptomatic nonunion without further need for surgical treatment. In 17 patients radiological follow-up ended after 6 weeks because they were absolutely pain free, had a ROM of at least 80% compared to the unaffected shoulder and no further follow-up was wished by the patients. Five patients were lost to follow-up. In a prospective, multicenter clinical trial, 2 patients out of 67 patients who were treated with plate fixation for clavicular midshaft fractures showed nonunion [6]. It is reliable to our results.

This study should be interpreted in light of its potential limitations. Due to the fact, that it is a retrospective study, the data were collected from patient records and the clinical follow-up is not completed for 1 year postoperative in every patient so that some data had to been collected by a phone call. For this reason, subjective shoulder scores, i.e. DASH or Constant score, were not determined. The clinical follow-up was incomplete, and 5 patients were lost to follow-up. Additionally, the radiological follow-up was not performed for every patient. Many patients had a free ROM and no pain at the fracture site during the 6 weeks follow-up examination was found, so no further radiological follow-up was performed, and fracture healing was clinically assumed. Furthermore, it is an uncontrolled study with no control group of our patients treated by VariAx plate fixation for clavicle fractures. Therefore, we relied on comparison with existing literature. All VariAx plates we implanted are superior plates. Plate discomfort risk could decrease by using anterior VariAx plates^21^, whereas fracture rigidity and stiffness was reduced with anterior plating [9].

## Conclusions

With this new pre-shaped locking plate, good to excellent intraoperative fit to the anatomical shape of the clavicle can be achieved. The implant seems to be reliable regarding handling and complications. Clinical and radiological results were very good, and a low complication rate was reported. The hardware removal rate was lower than those reported for non-contoured plates, but higher to other studies using pre-contoured plates although plate discomfort rate is comparable. The decrease of hardware-related plate removal might favor the use of pre-contoured clavicle plates.

## Abbreviations

a.-p.: antero-posterior
ASA: American Society of Anesthesiologists
CI: Confidential
CT: Computed tomography
DASH: Disability of the arm, shoulder and hand
NSAID: Non-steroidal anti-inflammatory drugs
OR: Odds ratio
ROM: Range of motion
SD: Standard deviation interval
